# A rapid evaluation of acute hydrogen sulfide poisoning in blood based on DNA-Cu/Ag nanocluster fluorescence probe

**DOI:** 10.1038/s41598-017-09960-1

**Published:** 2017-08-29

**Authors:** Yanjun Ding, Xingmei Li, Ceng Chen, Jiang Ling, Weichen Li, Yadong Guo, Jie Yan, Lagabaiyla Zha, Jifeng Cai

**Affiliations:** 1 Department of Forensic Science, School of Basic Medical Sciences, Central South University, Changsha, 410013 Hunan P.R. China; 2 Department of Pathophysiology, School of Basic Medical Sciences, Central South University, Changsha, 410013 Hunan P.R. China

## Abstract

Hydrogen sulfide (H_2_S) is a highly toxic gas as a cause of inhalational death. Accurate detection of H_2_S poisoning concentration is valuable and vital for forensic workers to estimate the cause of death. But so far, it is no uniform and reliable standard method to measure sulfide concentrations in H_2_S poisoning blood for forensic identification. This study introduces a fluorescence sensing technique into forensic research, in which a DNA-templated copper/silver nanocluster (DNA-Cu/AgNCs) fluorescence probe has been proposed to selective detection of S^2−^. Under an optimized condition, the proposed method can allow for determination of S^2−^ in the concentration range of 10 pM to 1 mM with a linear equation: y = −0.432 lg[S^2−^] + 0.675 (R^2^ = 0.9844), with the limit of detection of 3.75 pM. Moreover, acute H_2_S poisoning mouse models were established by intraperitoneally injected different doses of Na_2_S, and the practical feasibility of the proposed fluorescence sensor has been demonstrated by 35 poisoning blood samples. This proposed method is proved to be quite simple and straightforward for the detection of H_2_S poisoning blood. Also it may provide a basis ﻿for sulfide metabolizing study in body﻿, and it woul﻿d be m﻿eaningful to further pus﻿h﻿ forensic toxicology identification and clinical laboratory research.

## Introduction

Hydrogen sulfide (H_2_S), known as a colorless, flammable and water-soluble gas with the distinctive smell of rotten eggs, has drawn attention as an environmental hazard for many decades. It can be contacted in the oil and gas industry, agriculture, sewage and faeces treatments, infrastructure construction including asphalt operations, marshy terrain development and other settings^1^. Even worse, suicide by H_2_S inhalation is an emerging trend in some countries^2^. So it’s no strange that H_2_S is a highly toxic gas-second only to carbon monoxide as a cause of inhalational deaths^3^. Studies have proved that H_2_S could disturb people’s eyes, respiratory system and central nervous system when inhaled at a low concentration. While at a high concentration, it could cause permanent brain damage, even death on account of a respiratory failure by binding with cytochrome oxidase^4, 5^. However, the current situation that H_2_S poisoning having few specific histopathological findings was often hard to be diagnosed, made a confine in the field of forensic identification, especially for the analysis of H_2_S as a supporting evidence to the diagnosis of H_2_S poisoning in forensic identification^6, 7^. Therefore, developing a simple, selective and sensitive detection method for H_2_S poisoning biosample is urgent and popular, no matter for the establishment of an accurate treatment for victim, or a qualitative and quantitative analysis for the poisoning death.

Currently, several methods have been used to measure H_2_S, including high pressure liquid chromatography (HPLC)^8^, methylene-blue and gas chromatography/mass spectrum (GC/MS)^9^ and electrochemical sensors^10^. However, there are some disadvantages in detection system, respectively. Method based on HPLC was limited in the application of biosamples with the lack of capability to a real-time measurement^11^. Methylene-blue and GC/MS, which may be the most widely used method for the detection of H_2_S, but their detection sensitivities are low at concentrations(>1 µM) and need long incubation time (with more than 30 min) and sophisticated pretreated procedures^12^. In spite of electrochemical sensor providing high sensitivity, it needs to overcome the difficulties in removing harmful substances and proteins during detection^13^.

Recently, great attention has been paid to fluorescence sensor due to its simple operation, high selectivity and sensitivity compared with the above methods. Various metal materials including gold, silver and copper have been widely used to construct fluorescence systems to detect various analytes^14–16^. Recently, DNA-templated metal nanoclusters (NCs) fluorescence probes have enormous advantages, such as excellent photostability, good biocompatibility and sub-nanometer size. Thus, fluorescent DNA-metal nanoclusters  have extensively been used in biomedical application^17–19^. For instance, DNA-templated silver nanocluster probe was reported to detect in-solution multiplex miRNA^20^. Double-strand DNA (dsDNA) can act as a template for synthesis of CuNCs, which was used to distinguish the deletion or duplication genotypes^21^. These performances really make metal nanoclusters as a strong candidate for bio-sample rapid detection.

Due to the serious toxicological effects of H_2_S and the shortage of the current detection method, many researchers have developed different kinds of agents to deliver H_2_S in animal models^22–24^. Among the various researches, the sodium salts (NaHS and Na_2_S) were most widely and simply used to administer S^2−^ which was the major lethal component of H_2_S^25–27^. In this study, we choose the DNA-templated copper/silver nanocluster (DNA-Cu/AgNCs) as a fluorescence probe to develop the selective fluorescence sensor for rapid detection f S^2−^ in the H_2_S poisoning blood. DNA was used as the template for synthesis and stabilization of copper/silver bimetal nanoclusters. We then employed the DNA-Cu/Ag NCs to selectively detect S^2−^ on the basis of their specific interactions between Cu^2+^/Ag^+^ ions and S^2−^. The selectivity of the DNA-Cu/Ag NCs toward S^2−^ were evaluated. The analytical characteristics of the as-prepared fluorescence sensor are discussed in detail, including the linear range and accuracy. Moreover, the practical feasibility of this fluorescence sensing system was further validated by detecting biosamples of H_2_S poisoning blood, and the satisfied results were obtained.

## Materials and Methods

### Chemicals and apparatus

AgNO_3_(99.99%), NaBH_4_(98%), CuNO_3_(99.99%), Na_2_S, NaOH, NaSiO_3_, Na_2_CO_3_, NaCl, NaClO_4_, NaF_4_, NaHCO_3_, NaHSO_4_, NaNO_3_, CH_3_COONa, Na_2_SO_3_ and NaHSO_3_ were obtained from Sigma-Aldrich. The DNA sample (5′-CCCTTAATCCCC-3′) was separately purchased from Shanghai Sangon Biotechnology Co. Ltd. (Shanghai, China). Adult male Kunming (KM) rats (20 ± 2 g) were obtained from Shanghai Laboratory Animal Co. Ltd. Other reagents were of analytical grade and used without further purification.

All fluorescence measurements were recorded on a fluorescence spectrophotometer (Hitachi, F-4600) using a 350 μL quartz cell. The emission spectra of DNA-Cu/AgNCs were recorded from 500 nm to 675 nm. Both the emission and the excitation slits were set to 10 nM, the scanning speed was 240 nm min^−1^. The UV−vis absorption spectra were obtained on an UV−vis spectrophotometer (Shimadzu, UV-2450). The prepared DNA-Cu/AgNCs was analyzed by using a Titan G2 60-300 transmission electron microscope (TEM, FEI, USA). FT-IR spectrograms were received by a Nicolet 6700 FT-IR spectrometer & microscope. The gas chromatography/mass spectrum (GC/MS) experiments were carried out with GC/MS system (Agilent, 7890B/5977 A).

### Establishment of H_2_S poisoned mouse model and sample preparation

To establish the H_2_S poisoned mouse model, we used the Na_2_S as a donor. Prior to conducting the experiments, a pre-test was carried out in order to increase the accuracy and efficiency of experiment. We set a series of Na_2_S with different doses (2 mg, 3 mg, 4 mg, 5 mg, 6 mg) based on other reports to find the precise lethal concentration of S^2−^ ^27, 28^. By using intraperitoneal injection technique, every mouse body-weight about 20 g was randomly injected with different dose Na_2_S mentioned above diluted by 0.1 mL normal saline. And we found the lethal dose here was about 6 mg and all the mice would die in 3 minutes after injected.

**Figure 1:**
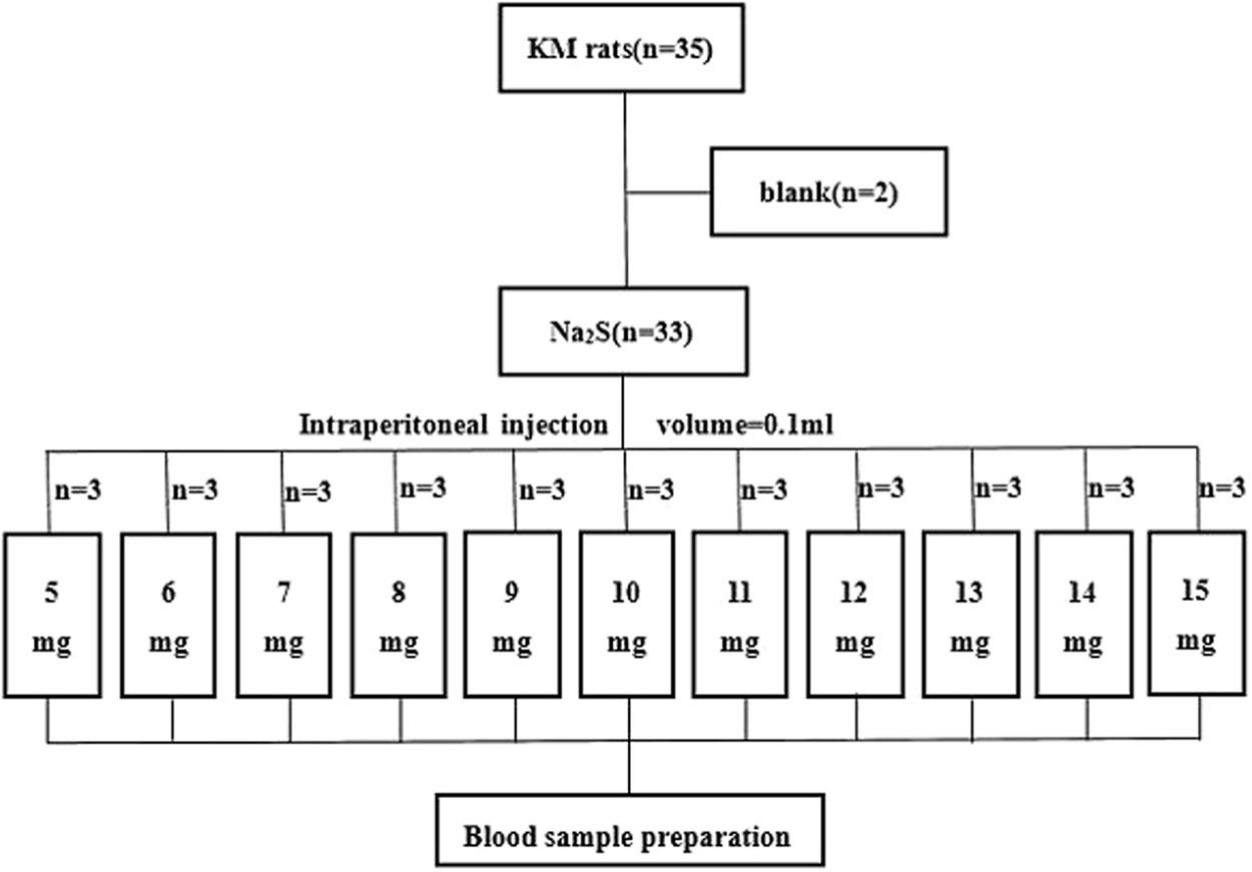
Experimental design for blood samples of H_2_S poisoning.

In the subsequent experiment, the H_2_S poisoning mouse model was established (Fig. 1 **)**. 35 mice from the same batch whose weight about 20 g were randomly divided with two groups: model group (n = 33) and control group (n = 2). Different doses of Na_2_S (5 mg, 6 mg, 7 mg, 8 mg, 9 mg, 10 mg, 11 mg, 12 mg, 13 mg, 14 mg, 15 mg) were injected intraperitoneally to mice from the model groups. Each reaction dose set three parallel experiments with the same handling. Finally, blood samples were collected by removing eyeball method into heparinized 1.5 mL polythene tubes 3 minutes later and sealed preservation at −4 °C until analysis.

### Synthesis of DNA-Cu/AgNCs probes

The DNA-Cu/AgNCs probes were prepared according to the previous literature procedure^29^. Briefly, 45 μL of 1 mM AgNO_3_ and 45 μL of 1 mM CuNO_3_ were added into 100 μL of 50 μM DNA and mixed together sufficiently. After put in an ice bath for 15 min, 30 μL of 2 mM NaBH_4_ was quickly added into the mixture, and the final solution was incubated at indoor temperature in the dark for 2 h before use.

### Fluorescence detection of S^2−^ using DNA-Cu/AgNCs probes

To develop this analytical method based on fluorescence sensing between DNA-Cu/AgNCs probes and S^2**−**^, the optimal conditions, including the pH and reaction time, were conducted anteriorly. The fluorescence reactions between 30 μL of 100 nM Na_2_S and 50 μL of DNA-Cu/AgNCs probes were detected at different pH values from 5.5 to 8.0 and different reaction time from 1 min to 12 mins for the optimal response conditions, respectively. Subsequently, fluorescence spectra were recorded to verify the quantitative, selective and biocompatible ability of DNA-Cu/AgNCs probes. On the one hand, 30 µL of Na_2_S solutions with different concentrations from 1 pM to 50 mM were added into 50 μL of DNA-Cu/AgNCs probes solution in order to investigate the quantitative ability of probe. On the other hand, to judge the selectivity of the DNA-Cu/AgNCs probes, 10 μM of various sodium salts including NaOH, NaSiO_3_, Na_2_CO_3_, NaCl, NaClO_4_, NaF_4_, NaHCO_3_, NaHSO_4_, NaNO_3_, CH_3_COONa, Na_2_SO_3_, NaHSO_3_ and Na_2_S were added into DNA-Cu/AgNCs probes and the fluorescence responses were detected under the same conditions mentioned above. Finally, to investigate the practical feasibility, hydrogen sulfide poisoned mice blood samples from different lethal doses were diluted to 50 folds, where 30 μL diluted blood samples containing various H_2_S concentration were added into 50 μL of DNA-Cu/AgNCs respectively.

### Data Availability

The datasets generated during and/or analysed during the current study are not publicly available due to there is no corresponding database, but are available from the corresponding author on reasonable request.

## Results and Discussion

### Detection principle of S^2−^ based on DNA-Cu/AgNCs probes

**Figure 2:**
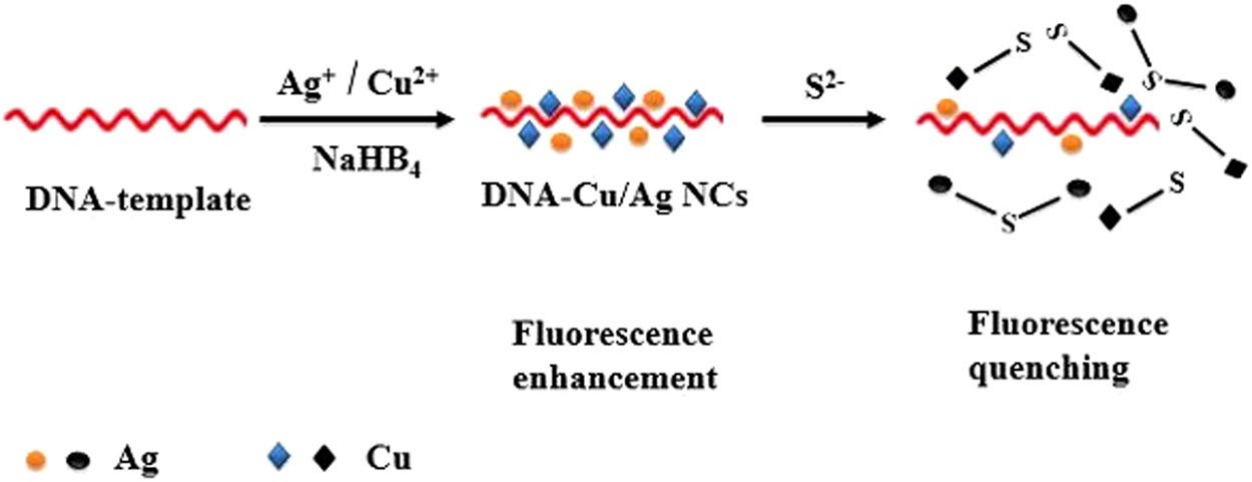
Schematic illustration of the fluorescence sensor for determination of S^2−^ based on DNA-Cu/AgNCs.

**Figure 3:**
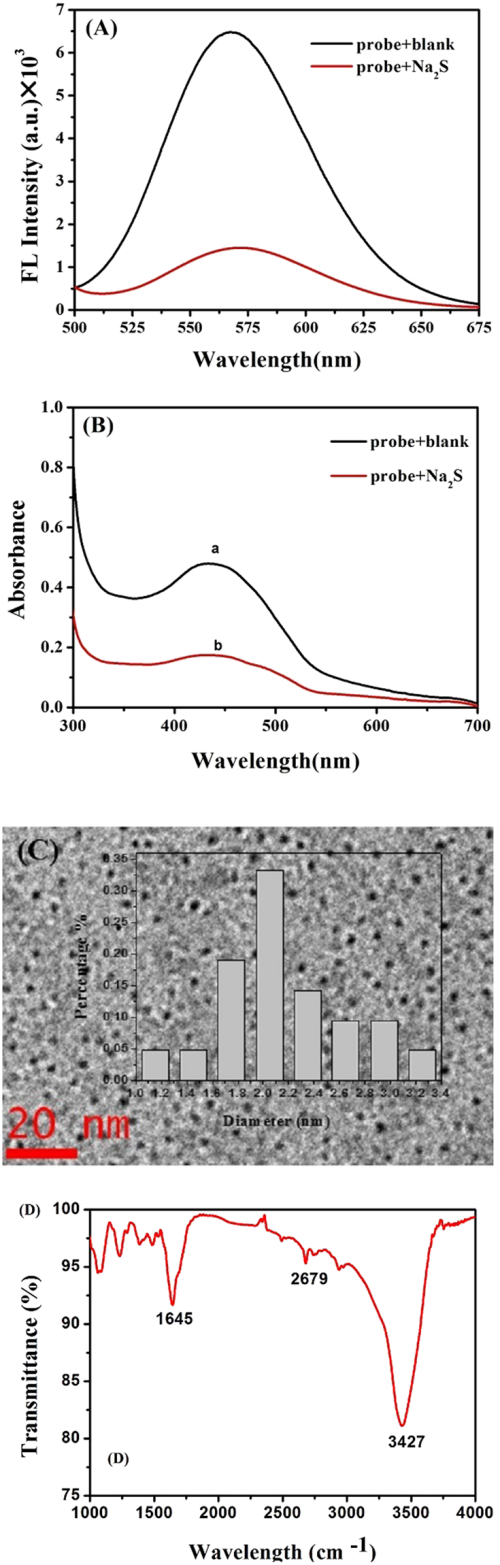
(**A**) Excitation wavelength of DNA-Cu/AgNCs and its corresponding fluorescence intensity in the absence and presence of Na_2_S. (**B**) UV- spectra recorded from DNA-Cu/AgNCs probes before (a) and after (b) the addition of 100 µM Na_2_S. DNA-Cu/AgNCs probes at pH 7.0 with 10 minutes’ incubation. (**C**) TEM image of the DNA-Cu/AgNCs, the inset showed the corresponding size distribution diagram. (**D**) FT-IR spectrum of DNA-Cu/Ag NCs.

The proposed fluorescence sensing mechanism for detection of S^2−^ was shown in Fig. 2. In this detection system, the DNA-Cu/AgNCs fluorescence probes were synthesized with DNA template (5′-CCCTTAATCCCC-3′)^29^. The proposed DNA sequence enriched in ‘C’ and ‘T’ may have affinity to the silver atoms and copper atoms, respectively, which has enabled the production of DNA-Cu/AgNCs with excellent fluorescence properties^30, 31^. As shown in the Fig. 3, the synthetic DNA-Cu/AgNCs probes displayed strong fluorescence emission at 568 nm when excited at 500 nm. Whereas, once a solution containing S^2−^ was introduced, the structure of fluorescence DNA-Cu/AgNCs probes was changed because sulfide ions have strong affinity with silver and copper atoms. The structural changes induced greatly quenching the fluorescence of DNA-Cu/AgNCs. Therefore, the concentration of S^2−^ can be detected quantitatively via the change of the fluorescence intensity detected by DNA-Cu/AgNCs.

### Characteristics of DNA-Cu/AgNCs probes

In order to get the information of DNA-Cu/AgNCs probe, four different techniques were used to characterized the DNA-Cu/AgNCs probe. Firstly, the fluorescence spectra of the prepared DNA-Cu/AgNCs were recorded from the spectrophotometer. As shown in Fig. 3A, we can distinctly achieve that the DNA-Cu/AgNCs probes have a strong fluorescence intensity of 6418 a.u. (excited at 500 nm), being in accordance with prior study^29^. Secondly, UV-vis spectra of DNA-Cu/AgNCs probes were recorded as Fig. 3B to further explore the mechanism that DNA-Cu/AgNCs probes worked as the model fluorescent probe for Na_2_S. As can be seen in Fig. 3B, the absorption peak at about 450 nm decreased after the addition of 30 μL of 100 μM Na_2_S. These results indicated that the fluorescence quenching of DNA-Cu/AgNCs probes was induced by the reaction between S^2−^ and DNA-Cu/AgNCs, according to the reports that S^2−^ has a strong binding affinity to copper and silver^32, 33^. Moreover, TEM and FT-IR of DNA-Cu/AgNCs probes have been investigated and showed in the Fig. 3C and D, respectively. As shown in Fig. 3C, the DNA-Cu/AgNCs were mostly spherical in shape and had an average size of 2.0 ± 0.2 nm without large nanoparticles or aggregation, which was in agreement with literature^34^. The surface chemistry of alloy NCs was confirmed by FT-IR spectroscopy (Fig. 3D **)**. The band at 3427 cm^−1^ corresponded to the -OH stretching and the band around 1645 cm^−1^ arose from the C = O stretching^35^. In addition, no S-H bond stretching appeared at 2568 cm^−1^, which implied that the sulfhydryl (-SH) has bounded to silver atoms and copper atoms to form a complex. These results indicated that DNA as the template protected the DNA- Cu/Ag NCs successfully^36^.

### Optimization of fluorescence response

**Figure 4:**
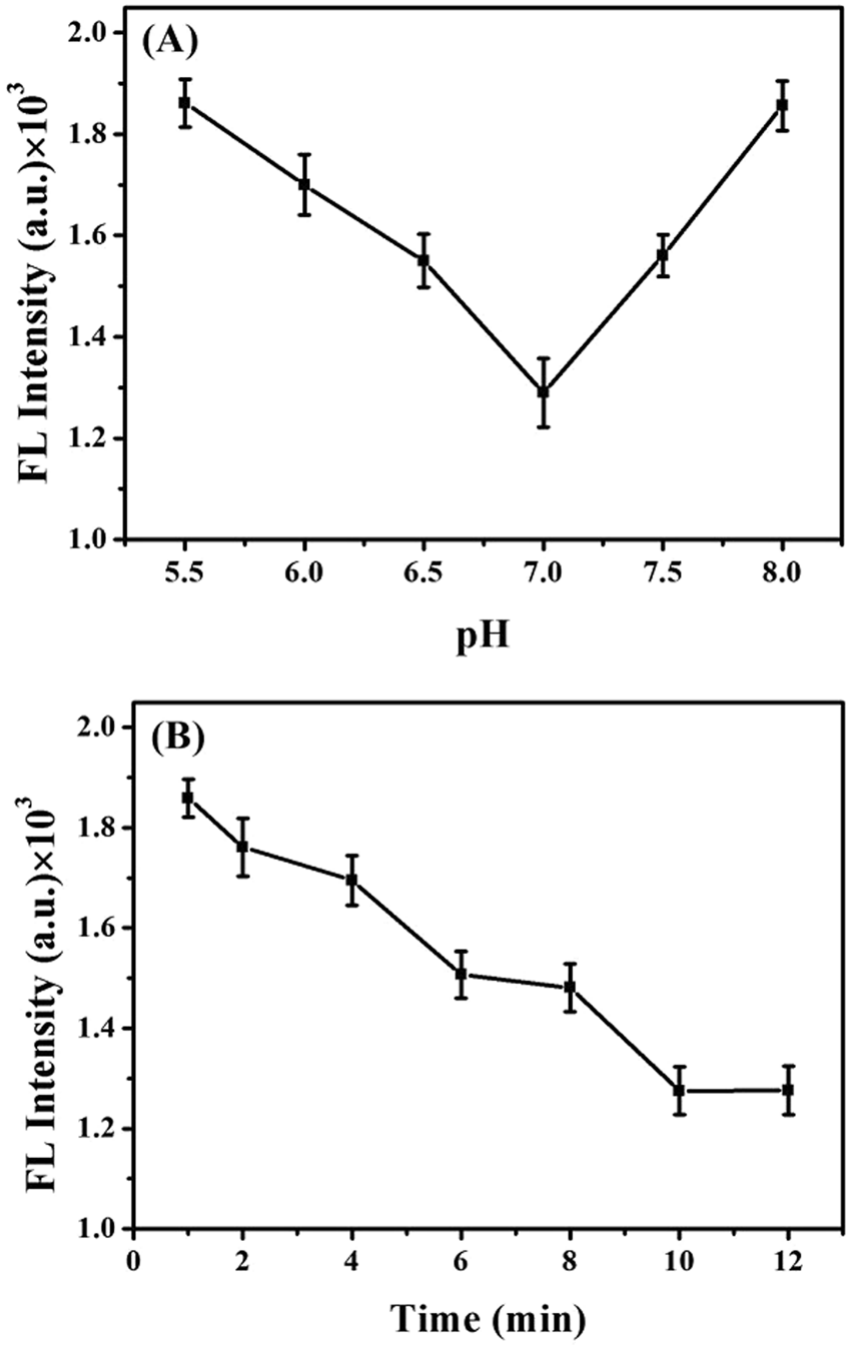
(**A**) pH dependence of the fluorescence sensor under the following conditions: 30 µL of 100 µM Na_2_S mixed with 50 µL of DNA-Cu/AgNCs at various pH valu﻿es from 5.5 to 8.0, incubated for 10 min at indoor temperature. (**B**) Reaction time dependence of the fluorescence sensor under the following conditions: 30 µL of 100 µM Na_2_S was added into 50 µL of DNA-Cu/AgNCs with different reaction time from 1 min to 12 min, incubated with pH 7.0 at room temperature.

For a precise study of the performance of DNA-Cu/AgNCs probes for the detection of S^2−^, the influences of pH value and incubation time in response system had been evaluated by the change of fluorescence intensity. As presented in Fig. 4A, the fluorescence intensities of DNA-Cu/AgNCs probes for Na_2_S had a strongest decrease at pH 7.0. Also, we can see from Fig. 4B that the fluorescence intensity apparently dropped to the lowest point at 10 minutes and held stable later. Therefore, pH 7.0 and 10 minutes of reaction time interval were selected as the optimal experimental conditions to attain the best effective utilization. These also probably implied that we didn’t need to prepare a special reaction condition or wait too long for a consequence based on this DNA-Cu/AgNCs probe, and it indeed made much sense when we run into an emergency and bad situation in practice.

### Selectivity of DNA-Cu/AgNCs probes

**Figure 5:**
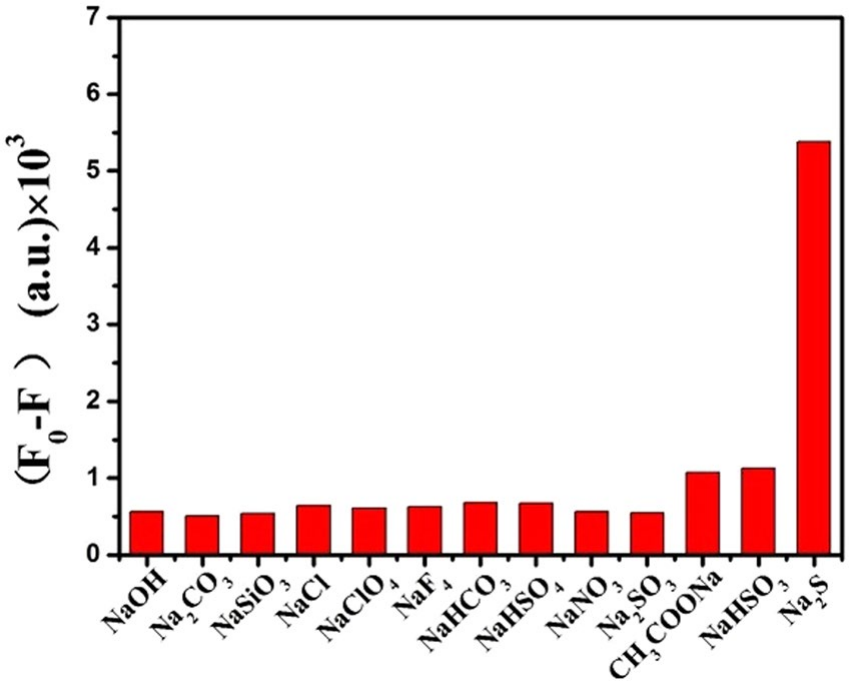
Selectivity of the probes was described through comparison of fluorescence intensity change of thirteen different anions, where F_0_ and F correspond to the fluorescence intensity of DNA-Cu/AgNCs in the absence and presence of analytical substances. All measurements were done in the same condition. 30 µL of various anions of 100 µM was respectively added into 50 µL of DNA-Cu/AgNCs.

Verification experiments of selectivity for S^2−^ were conducted over other twelve kinds of common cation ions including NaOH, NaSiO_3_, Na_2_CO_3_, NaCl, NaClO_4_, NaF_4_, NaHCO_3_, NaHSO_4_, NaNO_3_, CH_3_COONa, Na_2_SO_3_, NaHSO_3_ (Fig. 5). As shown from Fig. 5, the fluorescence intensity with the addition of S^2−^ decreased obviously, while all the other anion ions used above did not produce such obvious fluorescence reduction as much as Na_2_S in comparison. The fluorescence change (F_0_-F) of the sensor in the presence of Na_2_S is 5377 a.u., which was much higher than other ions, where F_0_ and F were the fluorescence intensity of the DNA-Cu/AgNCs solution without and with analytical substances, respectively. Such a phenomenon manifested that the DNA-Cu/AgNCs probe has good selectivity for S^2−^ free from strong interferences of various small molecules in common sample, which can make it a feasible sensor in complex samples.

### Quantitative analysis of S^2−^

**Figure 6:**
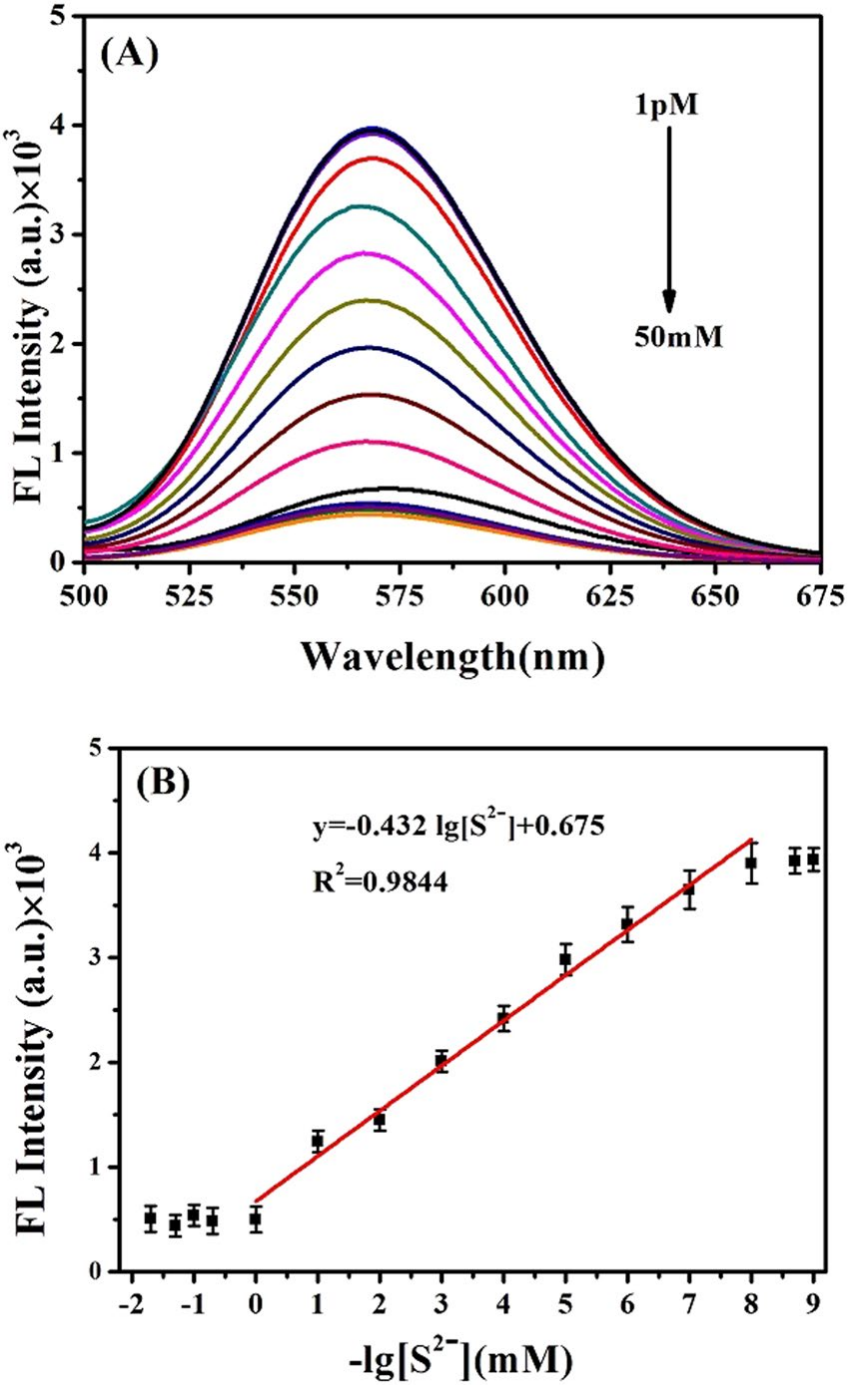
(**A**) Fluorescence spectra of DNA-Cu/AgNCs system after incubation with different concentrations of Na_2_S from 1 pM to 50 mM. (**B**) The linear relationship between the fluorescence intensity and concentration of Na_2_S. Each data point represents the average of the fluorescence responses of triplicate measurements.

Under the optimal conditions, the performance of this fluorescence sensing system was evaluated by adding various concentrations of Na_2_S (1 pM to 50 mM) to the DNA-Cu/AgNCs solution (Fig. 6). As illustrated in Fig. 6A, the result showed the fluorescence intensity decreased with increasing concentrations of S^2−^ from 10 pM to 1 mM. When the S^2−^ concentration was larger than 1 mM or smllar than 10 pM, fluorescence intensity kept a minimum or maximum value, and then reached a plateau. The plot of the fluorescence intensity as a function of the S^2−^ concentration was shown in Fig. 6B. The linear equation can be described as Y = −0.432 lg[S^2−^] + 0.675 (R^2^ = 0.9844). The limit of detection (LOD) was estimated to be about 3.75 pM, according to the 3σ (standard deviation) rule, which was much lower than the concentration reported in the previous studies^2, 6, 37^.

### Comparable analysis between fluorescence sensor and GC/MS

To provide a strong evidence for the feasibility and accuracy of the fluorescence sensor, a comparable analysis experiment for S^2−^ detection between the fluorescence sensor and GC/MS were conducted. Table 1 described the results and the relative deviations between the fluorescence sensor and GC/MS method. GC/MS is the most widely used method for H_2_S poisoning analysis recently and considered as standard in judicial identification, according to the profile of forensic technical specifications provided by the Ministry of Justice of the People’s Republic of China (SF/Z JD0107004-2010). As can be seen from the Table 1, the obtained relative deviations were in the range of −3.4–4%, which indicated both methods were in appreciable agreement. Therefore, the proposed fluorescence sensor may work as an alternative tool in the detection of H_2_S poisoning blood in forensic and clinical diagnostics.

**Table 1:** Comparison of S^2−^ levels determined using two methods with the proposed fluorescence sensor and GC/MS.

Samples	1	2	3	4	5
The proposed method (mmol·L^−1^)	0.062	0.078	0.081	0.086	0.098
GC/MS (mmol·L^−1^)	0.06	0.075	0.083	0.089	0.096
Relative deviation (%)	3.3	4	−2.4	−3.4	2.1

### Analysis of S^2−^ activity in mouse blood sample

**Figure 7:**
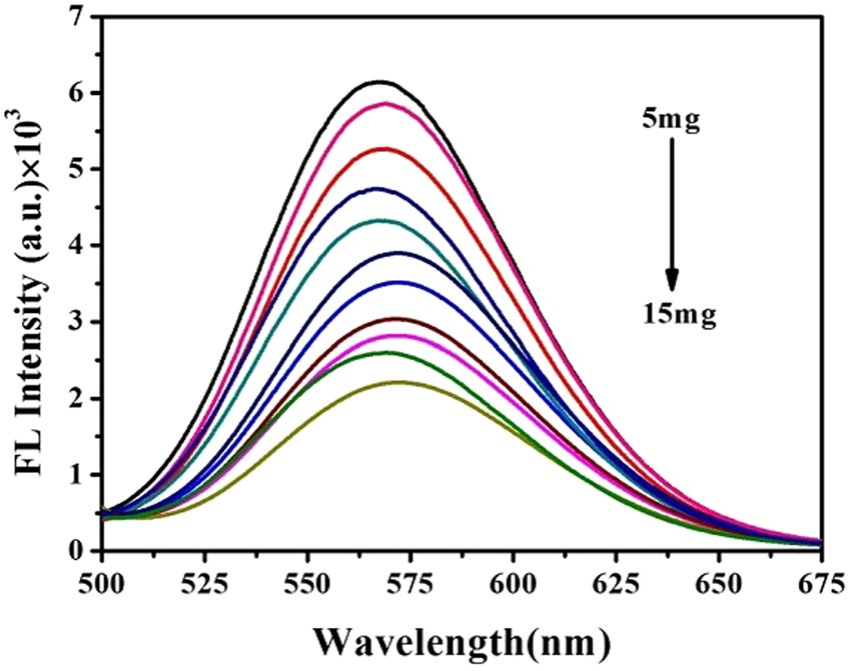
Biocompatibility of this proposed fluorescence sensor was conducted in H_2_S poisoning mouse blood samples, where mice were injected with 0.1 mL of different lethal dose from 5 mg to 15 mg and the blood samples were diluted to 50 folds. Then 30 µL of diluted blood samples of different concentrations were added into 50 µL of DNA-Cu/AgNCs probes.

In order to investigate the biocompability of the proposed fluorescence sensor, we established the H_2_S poisoning mouse models with intraperitoneal injection of different H_2_S concentrations and obtained 33 poisoning blood samples, which has been examined using the fluorescence sensor (Fig. 7). Excitingly, as shown in the Fig. 7, there was a corresponding decrease of fluorescence intensity with the increase of the S^2−^ containing in H_2_S poisoning blood, which illustrated that the proposed sensor system was pretty feasible in the detection application of poisoning blood. Because H_2_S played a significant role of pathological processes in poisoning death, the fluorescence sensor based on DNA-Cu/AgNCs probe can be a potential tool for the detection of H_2_S in blood samples for practical applications including estimation of time and death cause.

## Conclusions

In this work, a novel fluorescence sensor has been developed for rapid, simple and sensitive detection of H_2_S poisoning blood based on DNA-Cu/AgNCs. The DNA-Cu/AgNCs fluorescence probe, was very easyto synthesize and stable for a long-term storage. Under the optimal conditions, it provided a possibility for a quantitative analysis, and a linear equation y = −0.432 lg[S^2−^] + 0.675 (R^2^ = 0.9844) for 10 pM to 1 mM of S^2−^ was obtained, with limit of detection of 3.75 pM. Compared with existing methodologies, such as the official GC/MS method, this fluorescence sensor was quite simple and straightforward and can be completed in a few minutes without the need of any expensive equipment or trained personnel. Moreover, the probe was further used to monitor H_2_S poisoning blood, and results showed there was an obvious downward trend with the increase of concentration towards S^2−^, which confirmed its good biological application feasibility. Overall, the proposed fluorescence sensor method allows for favorable selectivity, high efficiency and sensitivity towards H_2_S poisoning blood. And it may offer a promising new detection approach for toxicant-metabolizing study in the forensic and clinical fields that makes significant social meanings.
